# Medicinal Plants in Brazil

**Published:** 1885-11

**Authors:** 


### Medicinal Plants in Brazil.

Consul Wright, of Santos, Brazil, incloses in a letter to the State Department notes upon the medicinal plants of that country. The compilation is the work of S. S. Schindler, a native-born citizen of the United States, who is now in Brazil. From Mr. Schindler's notes it appears that the country abounds in herbal remedies, and that alvelos, the new cancer cure, is but one of hundreds of trees possessing properties of great value, as yet almost unknown to materia medica.

